# India Cholera Statistics*Report on the Cholera Epidemic of 1868, in the Central Provinces. Folio, pp. 85. 1869.

**Published:** 1871-08

**Authors:** S. C. Townshend

**Affiliations:** Sanitary Commissioner, Nagpore


### Indian Cholera Statistics.*

By S. C. Townshend, M. D., Sanitary Commissioner, Nagpore.

Dr. Townshend arrives at the following conclusions from the documentary evidence in the body of his report:—

1. That for the production of cholera two conditions are necessary—the presence of a special contagion, and a susceptibility to its influence on the part of the person to whom the contagion is applied.

2. That with respect to the origin of the epidemic of 1868, the evidence is in favor of the contagion having been brought from elsewhere, rather than that it was generated in the localities where the disease first broke out.

3. That the subsequent diffusion of the contagion was effected solely by means of human intercourse.

* Report on the Cholera Epidemic of 1868, in the Central Provinces. Folio, pp. 85. 1869.

4. That a high temperature and extreme dryness are no obstacles to the diffusion of the contagion.

5. That with respect to the general population of the country the imbibition of water containing animal organic impurities is the most common means by which personal susceptibility to the effects of the contagion is induced.—*Half-Yearly Abstract.*

